# Why Minor Gynecological Operations Fail to Give Anticipated Results1Read before the section of Obstetrics and Gynecology of the Buffalo Academy of Medicine, March 24, 1903.

**Published:** 1903-07

**Authors:** Sigmund Goldberg

**Affiliations:** Buffalo, N. Y., Gynecologist, German Hospital; Consulting Gynecologist, German Hospital Dispensary. 508 Eagle Street


### Why Minor Gynecological Operations Fail to Give Anticipated Results.[1]

By SIGMUND GOLDBERG, M. D., Buffalo, N. Y.,
Gynecologist, German Hospital; Consulting Gynecologist, German Hospital Dispensary.

AT THE outset I would define a minor gynecological operation as one which does not involve the opening of the peritoneal cavity.

We may accept as an established axiom that the majority of genital ailments are due either to trauma during accouchement, or specific infection, and that the ultimate results of such accident or infection, in the course of time, will produce certain pathological conditions irreparable other than by operative procedure.

The ultimate results may be stated to be diseases of the uterine adnexa, which are not to be relieved by treatment, surgical or medical, on the original lesion. For example: given an injury of the cervix during labor, the same not being repaired at time of injury, or within six months or one year following, it is but natural that we should expect a development of an endometritis due to eversion of the contiguous mucous membrane, and it is

1. Read before the section of Obstetrics and Gynecology of the Buffalo Academy of Medicine, March 24, 1903.

but natural to infer, further, from the history of numberless similar cases, that such contiguous disease will not so remain, for there is no structural barrier to so limit the process, the mucous membrane of the cervix, uterus and fallopian tubes being of very similar micro- and macroscopical construction.

Nevertheless, it seems that nature, ever judicious in her ends, has formed some protective barriers, which are effective under ordinary conditions of health. For instance, Wyder says, that the epithelium on the crests of the endometrial folds is usually described as having cilia, which, Wyder insists, have a motion from the os internum toward the fundus.

Hofmeier (*Centralblatt für Gynækologie*) criticises this view. Not only were his own studies conducted upon fresh uteri, removed from mammals in which the conditions ought to be the same as in the human female, but he also examined organs removed at the operating table and at once immersed in a warm saline solution. In several of these latter he demonstrated conclusively, by removing strips of endometrium and placing them under the microscope, that minute particles of charcoal were invariably carried by the ciliary movement from the fundus toward the os.

This observation of Hofmeier seems at least to be in harmony with an intelligent design of nature, by which obstacles are interposed to the easy invasion of the upper reaches of the genital tract. But under the stress of injury or infection, these frail barriers are no longer effective, or, at least, in a great measure, have lost their potency, and this once lost, progressive endometrial infection, traveling on and infecting the adnexa, is the next step in the progress of the majority of the sexual ills of womankind. Next we find, due to the constant irritation and consequent increased weight of the uterus, a complication of adnexial disease, a retroversion or flexion, or both; and this condition, in my humble opinion, I consider the most common complication of adnexial disease.

Therefore, this condition generally receives the most attention, because of its alleged influence on the general health; but to my mind these various retropositions of the uterus are, *per se*, insufficient to intelligently account for all the symptoms usually ascribed to them. True, they may give some feeling of discomfort, some difficulty of locomotion, and in many cases not even this. The more active symptoms, generally ascribed to retroposition, such as pain, metrorrhagia, sacralgia, and more or less permanent invalidism, are due to the adnexial involvements.

It seems to me the height of folly to repair the cervix and fasten the uterus by the Alexandrian method or any of its modifications. For, after one year, we may feel morally certain that the seeds of disease are sown in the tubes and ovaries, although we may not always be able to positively demonstrate the exact condition by the usual method of examination.

J. Sprecht says that the early stage of pyosalpinx or salpingitis is hard to recognise because it follows immediately upon, or is associated in conjunction with, endometritis, the endometritis masking the symptoms of tubal disease. It is on account of our diagnostic fallibility that so many minor gynecological operations, such as the Alexander, curetage and trachelorrhaphy, result with only a modicum of benefit, and the cause, therefore, being the failure to recognise the early stages of disease in the adnexa. It was this very fact that induced A. Goldspohn, of Chicago, in his classic article published in the *Medical Record* of October 8, 1898, to advocate opening the internal inguinal rings for the purpose of inspecting the uterine adnexa during the performance of an Alexander operation.

The idea of opening the rings and inspecting the ovaries in every case, to ascertain their condition, was first impressed on Prof. Goldspohn, on September 18, 1893, during the performance of an Alexander operation, and not being able to find one of the round ligaments, he opened the internal inguinal ring and traced the ligament from the uterus within, outward. Incidentally the tubes and ovaries were discovered to be badly diseased and were taken out. A good cure, anatomically and subjectively, was obtained and the serious mental derangement, which had prevented a careful examination and exact diagnosis, disappeared entirely. Since then he has made a practice of introducing a finger and examining the adnexa very generally, in connection with the operation on the ligaments, which can really never be duly drawn out and shortened sufficiently without opening the peritoneal cavity.

I call your attention to what he says, further, regarding the practical importance of opening occluded tubes, destroying cystic follicles in ovaries or removing diseased parts from them, or of securing them in an approximately normal position. Goldspohn was not convinced until more recent years, when clinical experience, chiefly with patients after the Alexander operation, forced him to accept it as a general principle, that in cases of displacement of the uterus, any operation for this alone is a fulfilment of scarcely half our duty, and that nearly an equal obligation remains for treatment at the same time of the adnexa, in a large percentage of cases.

I object to the Goldspohn method, because it punctures a hole in the abdomen, the manipulative power of the finger is cramped, and we have a hole through which to work, instead of an incision, and we do not have the benefit of sight into the abdomen. But I do approve of the general principles underlying Goldspohn's modification of the Alexander operation, accidentally discovered, and at the same time finding ovaries and tubes of whose diseased condition he was unaware and of which we can never be absolutely certain.

I further believe it to be a great error, as stated in the majority of textbooks, that the Alexander operation is applicable in freely movable retroposition. I have, in quite a number of cases, during my service at the German Hospital and in my private practice, demonstrated to several gentlemen present, the free movability of the uterus and the apparent healthfulness of the adnexa as far as physical examination could demonstrate. Yet, believing, in every instance from the long standing history of injury or infection, that the uterine adnexa must of necessity be involved, I have freely opened the abdomen and fully demonstrated their diseased condition.

I do not wish to be understood as advocating that the Alexander operation has no place in our efforts for the alleviation of suffering woman, but I do unreservedly claim that it should be limited entirely to acute retroversion of recent standing, such as may be caused by a fall or such as would not be likely to produce an infective condition of the genital organs; and even here, occasionally, we may be disappointed in results if case has been of long standing.

While I am well aware how tedious the detailing of cases is to practical men, I cannot refrain from reporting one in support of this:

Miss W., pupil nurse at the German Hospital, aged 33; first consulted me March 20, 1902, for sacralgia, metrorrhagia and a general feeling of invalidism. Receiving no benefit from various drugs prescribed by me, she submitted to an examination by Dr. Max Breuer, then on service at the German Hospital, and myself. The examination showed hymen intact and a retroverted uterus of third degree, and what appeared to be a peculiar ridge-like cicatrix in the vagina. No other abnormality or disease of adnexa could be discovered at this time. Alexander operation was proposed by us and accepted by Miss W. While under anesthesia, Dr. M. Hartwig, who was present and assisted, made a careful examination and agreed with me that he could find nothing which would invalidate the ordinary rules for performing an Alexander operation. It was found that the supposed cicatrix was a congenital atresia between the cervix and the anterior wall of vagina, pulling the cervix forward and throwing the fundus backward. I first performed the plastic operation for the separation of the cervix from the vagina, followed by an Alexander. I operated on one side, while Dr. Hartwig operated on the other. During her three weeks confinement to bed, Miss W. was very much relieved. After this she returned to her home in the country. Later a letter from her, saying that all symptoms had returned and she was no better than before the operation. I advised her by letter to return to Buffalo, which she did. On examination I found the uterus mechanically in excellent position, could feel the ovaries apparently not enlarged, but very sensitive to touch. I informed her that an operation on her ovaries would be necessary for her future relief. She gratefully declined this. I have since learned that some form of intraäbdominal operation was performed at the Rochester City Hospital, and that now she is in perfect health.

I have never yet had cause to regret opening the abdominal cavity when there was the least doubt in my mind as to the exact condition. On the other hand, a number of times I have had to regret when I was not sufficiently heroic, and allowed myself to believe that some tinkering gynecological procedure would be sufficient. In further substantiation of what I assert, I will quote from an article by Aug. Goelet, published in the *Medical Record*, August 29, 1896. He says, in summing up: "Alexander's operation is not necessary in movable retroversions unless they are complicated by prolapsed, enlarged and sensitive ovaries, which do not require removal."

Personally, I cannot conceive of a prolapsed, enlarged and sensitive ovary, which does not require some form of operative procedure for its relief, its prolapsed, enlarged and sensitive condition being generally due to a condition of cystic degeneration, a condition not amenable to any form of treatment except surgical, radical or conservative.

Goelet further claims that the operation of dilatation with curetage and the subsequent use of a glass drainage splint, is indicated in a large majority of cases of movable retrodeviation and is more rational, since it restores the normal position of the organ without submitting the patient to any risk and does not entail prolonged confinement to bed.

If Alexander's operation, *per se*, ever did any good, even under the rules laid down by its originator and his followers, why this threshing around among our ablest men in their attempts to improve or change it? Why do some of the leading gynecologists of the world, with their vast opportunities for observation, fail to record or announce any positive cure, unless a mechanical reposition for the uterus can be considered a cure, without taking into consideration the subsequent well being of the patient?

I will call your attention to a statement of Nicholas Senn, in his report of observations in Berlin, at the gynecological clinic of Prof. Olshausen. He says that Prof. Olshausen only commenced to perform this operation three years ago, and according to established rule limits operations to cases of retroversion of a movable uterus, which produces symptoms of sufficient severity to warrant operative interference. To this, again, I object, as the movability of uterus should not be taken as the sole indication for the performance of an Alexander operation.

But the most important point which I wish to bring out in connection with the report of Dr. Senn, is that of the wealth of material in this clinic, as shown by the fact that of about 700 patients suffering from retroversion of the uterus, applying annually for relief, only from 25 to 40 cases are selected for an Alexander operation. But Prof. Olshausen is as yet a new hand in the performance of this operation, and as his experience therewith increases with time, and he has had more opportunity for observation of its ultimate results, he will still further limit the number of his future operations.

Now compare with their wealth of material with regard to retroposition, the small number of Alexander operations performed in Europe, to the gigantic numerical statistics which can be exploited by American gynecologists. It seems to be the especial glory of every gynecologist, embryo or otherwise, to dilate on the number of Alexanders he has already performed, and, if opportunity is favorable, to demonstrate, as a result thereof, the more than normal position of the uterus. But, as their experiences keep pace with their advancement in years and for opportunities of observance of ultimate results, and their patients return to their offices and report themselves very little benefited, if any, by the operation, it will hardly soothe their mental dissatisfaction to tell them that the uterus is exactly where it ought to be or a little better placed.

I assert, then, that the ordinary trachelorrhaphy, curetage and Alexander operation, or the method of Goelet, by scraping and packing and his drainage splint, are insufficient to restore such a patient to complete health, and the failure to restore the patient is due to the fact that the time for such work is long past, unless the same be done within one year from time of infliction of original injury.

In conclusion, then, I would formulate for your kind consideration the following propositions:

1. That all so-called minor gynecological operations, to be of any permanent benefit, must be performed within at least one year from time of the infliction of the original lesion.

2. That after the passage of about a year, either Alexander's operation, trachelorrhaphy or curetage, or all combined, will be insufficient to restore patient to perfect health.

3. The reason for such failure in the great majority of such cases, after one year, is because the adnexa are sure to be involved.

4. The best course will be to do some operative procedure intraäbdominally, and not by the internal inguinal ring puncture of Goldspohn, but by free incision, so that the exact condition can be ascertained and properly treated, and this I hold to be proper rather than to depend upon the uncertainty of physical palpation.

508 Eagle Street.

